# Mineral Composition, Physicochemical Characteristics, and Antioxidant and Antibacterial Properties of Oil Extracted From Moroccan Bitter Apricot Kernels

**DOI:** 10.1155/sci5/7461290

**Published:** 2026-01-19

**Authors:** Mohamed Amine El Hajjaji, Kawtar Fikri-Benbrahim, Najoua Soulo, Younesse El-Byari, Zineb Benziane Ouaritini

**Affiliations:** ^1^ Department of Biology, Faculty of Sciences, Sidi Mohamed Ben Abdellah University, Fez, Morocco, usmba.ac.ma; ^2^ Department of Biology, Faculty of Sciences and Technologies, Sidi Mohamed Ben Abdellah University, Fez, Morocco, usmba.ac.ma

**Keywords:** antibacterial activity, antioxidant activity, bitter apricot kernel, Moroccan apricot, oil composition, *Prunus armeniaca*

## Abstract

Apricot kernels, often regarded as by‐products, are a valuable source of oils and bioactive compounds such as polyphenols, which have applications in pharmacology, the food industry, and cosmetology. This study aimed to address the research gaps regarding the nutritional and functional potential of Moroccan bitter apricot kernels (BAKs) by analyzing their mineral composition and evaluating the physicochemical, antioxidant, and antibacterial properties of their oil. The mineral composition of the kernels was analyzed using ICP‐AES. The physicochemical properties of BAK oil, including density (D), acid value (AV), saponification number (SN), iodine value (IV), refractive index (RI), and peroxide value (PV), were evaluated. The total phenolic content (TPC) and total flavonoid content (TFC) were estimated using the Folin–Ciocalteu and aluminum chloride colorimetric methods, respectively. The antioxidant potential was examined through 2,2′‐diphenyl‐1‐picrylhydrazyl (DPPH) radical scavenging and total antioxidant capacity (TAC) assays, and antibacterial activity was evaluated using disc diffusion and microdilution methods. Results showed that the apricot kernels are rich in essential minerals, with high amounts of potassium (184.75 ± 5.5 g/kg) and phosphorus (109.72 ± 2.5 g/kg). The physicochemical properties of the studied oil were as follows: D: 0.92 ± 0.001 g/cm^3^, AV: 0.65 ± 0.17 mg KOH/g of oil, SN: 187.15 ± 0.05 mg KOH/g of oil, IV: 95.5 ± 0.45 g I_2_/100 g of oil, RI: 1.470 ± 0.001, and PV: 0.98 ± 0.17 meq O_2_/kg of oil. The total phenolic and flavonoid contents were 41 ± 0.5 mg GAE/100 g and 23.1 ± 0.2 mg QUE/100 g of oil, respectively. The DPPH IC_50_ value was 6640 ± 0.32 μg/mL, and the TAC was 2.21 ± 0.12 mg AAE/g of oil. The highest antibacterial effect was observed against both Gram‐positive bacterial strains, with a MIC value of 100 μL/mL. This study demonstrates that BAKs are a noteworthy oil source, offering promising opportunities for applications in the food and pharmaceutical industries.

## 1. Introduction

Globally, large amounts of fruit by‐products are discarded each year, leading to disposal challenges [1]. Recently, they have gained attention for their richness in bioactive compounds such as polyphenols, with growing applications in food, agriculture, and animal feed. Notably, fruit seeds and kernels, due to their high oil content, represent valuable resources in the fruit processing industry [2].

Plant seed oils contain antibacterial and antioxidant chemicals [3, 4]. Oxidative stress is a pivotal factor in the development of several age‐related illnesses. As we age or when afflicted by diseases, there is a disruption in the equilibrium between the production and elimination of superoxide anions and potent free radicals. Introducing antioxidant compounds through supplementation can substantially mitigate the resulting damage. Therefore, taking steps to hinder the formation of free radicals within the body becomes crucial for safeguarding against a spectrum of diseases [5]. In addition to being reducing agents and chelators of pro‐oxidant metals, natural antioxidants also serve as singlet oxygen quenchers and free radical scavengers. They are extensively utilized in the food industry to improve food’s sensory appeal, nutritional value, or preserving quality of foods [6].

Antimicrobial resistance presents a multifaceted challenge, characterized by the emergence of microbial strains that have developed resistance to widely employed antimicrobial drugs and antibiotics. The bacteria examined in our current study, including *Escherichia coli*, *Pseudomonas aeruginosa*, *Staphylococcus aureus,* and *Bacillus subtilis*, have garnered attention in prior research due to their alarming levels of resistance [7–9].

The use of prunus species (apricot, peach, plum, and almond, among others) kernels, a waste by‐product, could have a positive economic impact and lessen waste disposal issues because these by‐products are produced in large quantities by the food canning industry [10, 11].

Apricot (*Prunus armeniaca* L.) is a Rosaceae family member and one of the world’s most important commercial crops [12]. Originally from China, the apricot plant was transferred and cultivated throughout Europe and the Mediterranean regions including Morocco. The largest apricot producers in the world, according to the Food and Agriculture Organization (FAO), are Turkey and Uzbekistan, with 833.398 and 529.109 tons of apricots produced in 2020, respectively, while Morocco is considered as the 10^th^ largest producer with a total production about 100.000 tons [13]. The pale yellow and tasteless oil extracted from apricot kernels can be utilized in a variety of applications, including cosmetic, edible, and industrial preparations. It serves as a significant source of both unsaturated and saturated fatty acids, such as oleic, linoleic, palmitic, myristic, palmitoleic, stearic, and linolenic acids [14, 15]. Agrotechnical practices, fruit variety, harvest year, and region of origin all affect the composition of apricot oil and nutrients [16]. Throughout history, both apricots and the oil derived from them have been employed to treat various ailments such as asthma, constipation, cough, vaginal infections, furuncles, acne vulgaris, dandruff, and skin irritation [17–19].

To the best of our knowledge, no published data have investigated bitter apricot kernels (BAKs) cultivated in Morocco, leaving a significant gap in understanding the nutritional and functional potential of this local resource. Therefore, the central hypothesis of this study is that Moroccan BAKs possess a distinctive mineral profile, while the oil extracted from them exhibits unique physicochemical properties and bioactivities (antioxidant and antibacterial), which may offer added value for food, pharmaceutical, and cosmetic applications. To test this hypothesis, the present study systematically evaluated the mineral composition of the kernels and the physicochemical characteristics and biofunctional activities of their oil.

## 2. Material and Methods

### 2.1. Chemicals

Aluminum chloride, ammonium molybdate, n‐hexane, methanol, Folin–Ciocalteu, butylated hydroxytoluene (BHT), quercetin, ascorbic acid, 2,2‐diphenyl‐1‐picrylhydrazyl (DPPH), sodium carbonate, gallic acid, and resazurin were purchased from Sigma Aldrich (Munich, Germany).

### 2.2. Plant Material

The apricot fruits, from *Prunus armeniaca* L. Maoui variety, were harvested from Sefrou region (33°40′13.6″N 4°51′28.7″W) in 2022. The plant was identified, and a specimen was lodged in the department of Biology, Faculty of Sciences, Sidi Mohamed Ben Abdellah University under code: RPA 001 VM 2326 SL. The kernels were manually separated and then ground into a fine powder using an electric blender (VEVOR Electric Grain Mill). The resulting powder was stored in a plastic bag at 4°C until use.

### 2.3. Mineralogical Composition Analysis

The mineral compositions of apricot kernels were analyzed with inductively coupled plasma atomic emission spectroscopy (ICP‐AES) (HORIBA JOBIN YVON) according to Sastre et al. [20]. Briefly, the sample was incinerated at 550°C to remove organic matrix that could be widely present. A volume of 10 mL of aqua regia solution prepared previously with concentrated nitric acid (HNO_3_) and of hydrochloric acid (HCl) (1:3 v/v) was mixed with 0.15 g of the obtained residue in a digestion vessel. The mixture was heated on a hot plate under a fume hood until approximately 90% of the solution had evaporated. After cooling, the digested solution was diluted to a final volume of 45 mL with 2M HCl and distilled water. The obtained solution was then filtered using Whatman No. 1 filter paper and analyzed using ICP‐AES. The mineral concentrations were calculated using equation (1):

(1) mineral concentration mgKg=concentrationmg/L×final volumeLsample weight g×1000.

### 2.4. Oil Extraction

The oil of apricot kernel was extracted, from powder (100 g), in a Soxhlet apparatus with n‐hexane (500 mL) for 12 h. A rotary evaporator was used to remove the solvent, and the obtained oil was stored at 4°C for further analyses. The yield of the obtained oil was 40.5%.

### 2.5. Physicochemical Properties

The physicochemical properties of the studied oil, including density, acid value, saponification number, iodine value, refractive index, and peroxide value, were determined using standard methods described by the AOAC [21].

### 2.6. Extraction of Phenolic Compounds

The phenolic compounds present in the studied sample were extracted using a liquid–liquid extraction (LLE) system according to Parry et al. [22]. Briefly, in a test tube, 1 g of BAK oil was added to 3 mL of methanol/water (80:20, v/v) solution and vortexed for 1 min. The supernatant’s recovery was done after 5 min of centrifugation at 6000 rpm. This procedure was repeated until a final volume of 10 mL was obtained.

### 2.7. Determination of Total Phenolic Content (TPC)

The Folin–Ciocalteu assay was employed to determine the TPC according to the method described previously by Amin et al. [23]. The sample (100 μL) was mixed with Folin reagent (500 μL) previously diluted (1/10), and four minutes later, 7.5% w/v sodium carbonate (400 μL) was added to the obtained mixture. The absorbance was measured at 760 nm after 2 h of incubation in the dark at room temperature. The total polyphenol concentration was calculated by making a calibration curve with gallic acid and was expressed as milligram of gallic acid equivalents per 100 g of oil (mg GAE/100 g of oil).

### 2.8. Determination of Total Flavonoid Content (TFC)

The TFC was determined using the aluminum chloride colorimetric method previously described by Bahorun et al. [24], with some modifications. Indeed, to 1.5 mL of the sample, 1.5 mL of 2% AlCl_3_ solution was added, and quercetin served as the reference standard. The mixture’s absorbance was measured at 430 nm, and the TFC was measured in milligrams of quercetin equivalent per 100 g of oil (mg QE/100 g of oil).

### 2.9. Antioxidant Activity

#### 2.9.1. DPPH Radical Assay

The stable radical species DPPH (4 mg DPPH/100 mL) has been utilized in this test according to the method outlined previously by Brand‐Williams et al. [25]. In an Eppendorf tube, 100 μL of the sample was added to 825 μL of the DPPH solution; the mixture was left in the dark for 30 min at room temperature. After the incubation period, the absorbance was measured at 517 nm. The inhibition percentage of DPPH was calculated using equation (2).

The value was then determined as half maximal inhibitory concentration (IC_50_), which represents the concentration required to scavenge 50% of free radicals in the reaction mixture.

(2) Inhibition %=absorbance control−absorbance sampleabsorbance control×100.

#### 2.9.2. Total Antioxidant Capacity (TAC)

To initiate the reaction, the reagent solution was prepared by mixing sodium phosphate (28 mmol/L), sulfuric acid (0.6 mol/L), and ammonium molybdate (4 mmol/L), and then 2.5 mL of this solution was added to 250 μL of the sample. The mixture was incubated for 2 hours in a water bath at 95°C and then left at laboratory temperature for 10 min before measuring its absorbance at 695 nm. The TAC of tested sample was expressed as milligram of ascorbic acid per gram of oil (mg AAE/g of oil) [26].

### 2.10. Antibacterial Activity

#### 2.10.1. Disc Diffusion Method

The antibacterial activity of the oil obtained from BAKs was evaluated using disc diffusion method as descripted by Muharni et al. [27]. In brief, 90 mm Petri dishes were seeded with 0.1 mL of a bacterial suspension, adjusted to 10^8^ colony‐forming units (CFU)/mL for each strain. After wells were made in the agar plates using a 6‐mm cork borer, 100 μL of the studied sample was pipetted into each well individually. The inhibition zone diameters were measured after 24 h of incubation at 37°C. Kanamycin and distilled water served as positive and negative controls, respectively. Tests were repeated at least three times at different intervals.

#### 2.10.2. Minimum Inhibitory Concentration (MIC)

The microdilution method was employed using the 96‐well microplate to determine the MIC of the tested oil [27]. Briefly, 50 μL of Luria–Bertani (LB) broth medium was transferred in each well except those of the first column which were filled with 100 μL of the tested sample. Subsequently, serial microdilution was performed to obtain a concentration of the tested sample ranging from 100 to 0.024 μL/mL. Next, each well has received 50 μL of the bacterial suspensions containing 10^6^ CFU/mL. Ampicillin and kanamycin served as positive controls, while the negative control received no tested sample neither cell suspensions. After incubation of 24 h at 37°C, 15 μL of sterile resazurin was added to each well, and the results were recorded after an additional two‐hour incubation.

The minimum bactericidal concentration (MBC) was determined by subculturing wells showing MIC‐level growth onto Mueller–Hinton agar and incubating under the same conditions. Experiments were performed in triplicate.

### 2.11. Statistical Analysis

Statistical analysis was performed using GraphPad Prism version 8.0 for Windows. Data are presented as means ± standard deviation (SD). The Student’s *t*‐test was used when comparing two groups, whereas one‐way ANOVA followed by Tukey’s post hoc test was applied for comparisons involving three or more groups.

## 3. Results and Discussion

### 3.1. Mineral Compositions

Minerals are important micronutrients in apricot. Potassium is a necessary nutrient that is vital to many biological functions, such as preserving osmolality and fluid equilibrium in cells [28]. Adequate calcium intake has been linked to increased bone mineral accumulation in early life and the prevention of osteoporosis and colorectal cancer. Additionally, it is essential to the heart’s electrical activity and pumping function [29]. Magnesium is one of the most overlooked minerals in the human body, yet it is essential for a healthy and long life. It activates over 300 enzymes, supports muscle and nerve function, and helps maintain a normal heart rhythm. Additionally, magnesium bolsters the immune system, regulates blood sugar levels, and aids in maintaining normal blood pressure [30].

**Figure 1:**
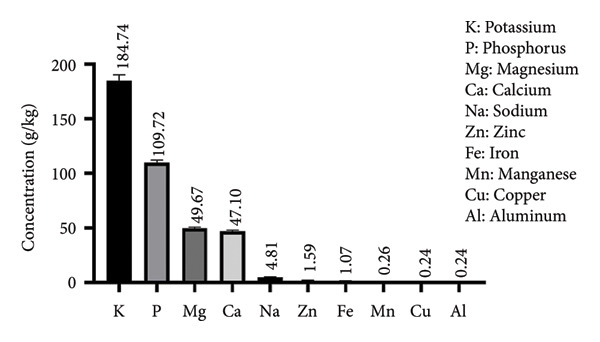
Mineral composition of bitter apricot kernels.

Results of apricot kernel samples analysis, using ICP‐AES, are presented in Figure 1. Significant concentrations of various essential minerals were observed, and the most prevalent minerals identified were potassium (184.749 g/kg), phosphorus (109.725 g/kg), magnesium (49.674 g/kg), and calcium (47.103 g/kg). On the other hand, aluminum, copper, and manganese were found to be the minor minerals with values of 0.242, 0.246, and 0.260 mg/kg, respectively.

The results found are aligned with those published by Alpaslan et al. [14], who found that BAKs contain several essential minerals, with particularly high amounts of potassium and magnesium, approximately 570 mg/100 g and 290 mg/100 g of dry weight, respectively. Furthermore, Bachheti et al. [15] reported the presence of various important minerals in seed oil in varying levels, including copper, potassium, calcium, phosphorus, iron, and magnesium. Another previous study conducted on several apricot cultivars from Turkey revealed that the mineral elements varied widely depending on the specific apricot cultivar kernels. All apricot cultivar kernels have high levels of K, P, Mg, Ca, and Na [31]. The differences in minerals across cultivars can be caused by diverse variables, including varieties, growing circumstances, harvesting time, genetics, regional variances, soil qualities, and analysis processes.

### 3.2. Physicochemical Properties

The physicochemical properties are shown in Table 1. The obtained oil was pale yellow and free of sediments. The density of our sample was found to be 0.92 ± 0.001 g/cm^3^, which is consistent with previously reported values [31]. An indication of an oil’s viability for industrial application is its acid value. Oils with an acid value below 2 mg KOH/g are typically considered desirable for ensuring good quality [32]. Our sample’s acid value was 0.65 ± 0.17 mg KOH/g of oil, indicating a relatively low level of free fatty acids and a good stability. Saponification value of the studied sample was 187.15 ± 0.05 mg KOH/g of oil. The saponification value compares the relative molecular weights of oils and determines their industrial applicability. It is determined by the average molecular weight (or fatty acid chain length) of all fatty acids present. Higher saponification values indicate that the oils have lower molecular weights [33]. The iodine value of the studied oil was 95.5 ± 0.45 g I_2_/100g of oil, the refractive index was 1.470 ± 0.001 at 20°C, and the peroxide value was found to be 0.98 ± 0.17 meq O_2_/kg of oil, which indicate that the studied oil has a good oxidative stability. Our results are in concordance with those reported by Shariatifar et al. [34], who investigated the physicochemical properties of four Iranian apricot kernel oils and found comparable values for iodine value (90.3–102.4 g I_2_/100 g oil), saponification number (183.3–195.5 mg KOH/g oil), peroxide value (0.35–1.9 meq O_2_/kg oil), and acid value (0.2–0.6 mg KOH/g oil). Likewise, studies on wild apricot kernel oils from India have shown similar values to those obtained in our work, with a saponification value of 123.4 mg KOH/g oil, an iodine value of 96.39 g I_2_/100 g oil, a refractive index of 1.468, and an acid value of 38.6 mg KOH/100 g oil [35]. Previous research has highlighted the impact of extraction methods on the physicochemical properties of almond oils. It was reported that almond oil extracted using the cold press method had higher acid values (2.21 mg KOH/100 g of oil), iodine value (13.76 mg/g), and saponification value (103.6 mg KOH/g of oil). In contrast, almond oils obtained by Soxhlet extraction showed a higher peroxide value (6.44 meq O_2_/kg) and refractive index (1.484 nD). These differences may be attributed to the effect of the extraction method [36]. According to the obtained results, the studied oil may be suitable for industrial, cosmetic, and food applications.

**Table 1:** Physicochemical properties of bitter apricot kernel oil.

Physicochemical properties	Values
Density (g/cm^3^)	0.92 ± 0.001
Acid value (mg KOH/g of oil)	0.65 ± 0.17
Saponification number (mg KOH/g of oil)	187.15 ± 0.05
Iodine value (g I_2_/100 g of oil)	95.5 ± 0.45
Refractive index	1.470 ± 0.001
Peroxide value (meq O_2_/kg of oil)	0.98 ± 0.17

*Note:* Results are presented as means ± SD.

### 3.3. Total Phenolic and Flavonoid Compounds

Polyphenols are natural compounds found in plants and are well known to have several biological activities such as antioxidant, anti‐inflammatory, and antibacterial [37]. The obtained results indicated that the TPC of our sample was 41 ± 0.5 mg GAE/100 g of oil (Figure 2(a)) and the TFC was 23.1 ± 0.2 mg QUE/100 g of oil (Figure 2(b)). These results are higher than those mentioned by Makrygiannis et al. [38], who found that the TPC recorded in the apricot kernel oil harvested in Greece in 2021 was 0.794 ± 0.23 mg GAE/100 g dw. In addition, previous research carried out by Sara et al. [39] on nut oils showed that their concentration of polyphenols varied from 8 to 70 mg GAE/100g of oil. Furthermore, the study conducted by Hamid et al. [40] reported that the TPC and TFC of BAK oil were 1060 ± 132 mg GAE/100g and 475 ± 11 mg QUE/100g, respectively. On the other hand, several studies which investigated the TPC and TFC in apricot kernel extracts reported that they presented high level of both these components when compared to those of apricot kernel oil [41–44]. Previous study was carried out by Dulf et al. [45], who highlighted the effect of solid‐state fermentation process (SSF) on the evaluation of TPC and TFC levels of apricot pomaces with *A. niger* and *R. oligosporus*. According to reports, *R. oligosporus* increased the levels of TPC and TFC by over 70% and 38% for SSF, respectively.

Figure 2Total phenolic contents (a) and total flavonoid contents (b) of bitter apricot kernel oil.

**Figure:**
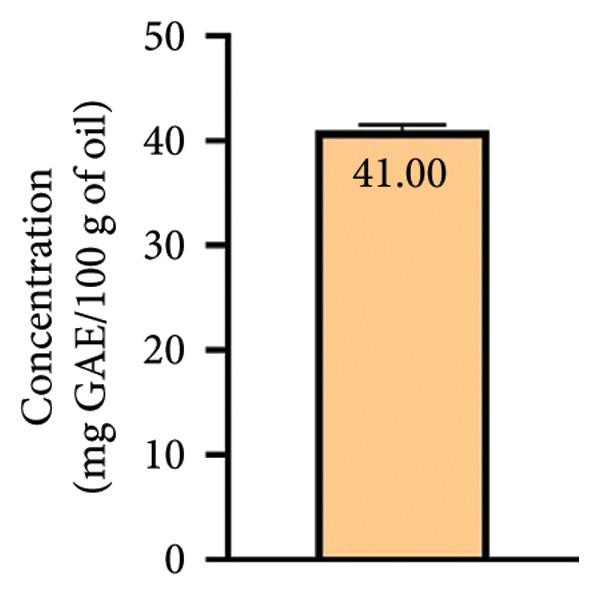
(a)

**Figure:**
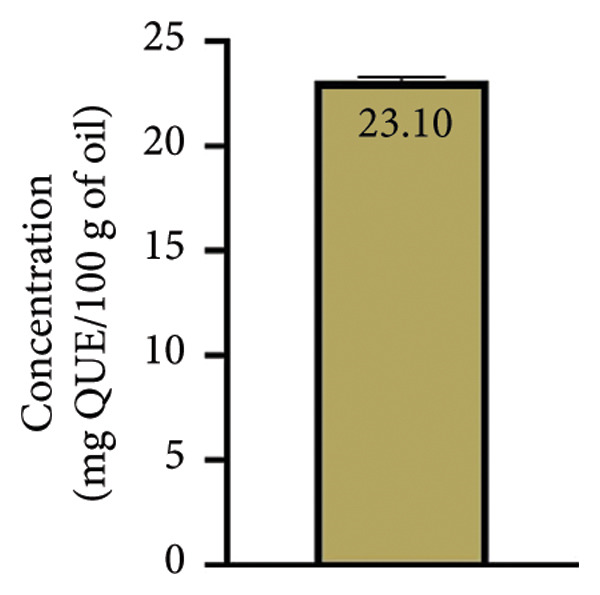
(b)

The limited concentration of the studied oil within the aforementioned compounds results in only marginal antioxidant attributes, as supported by the outcomes of both the DPPH and TAC assays. These findings underscore the relatively modest impact of apricot kernel oil in enhancing the compounds’ overall antioxidant efficacy. As per the research conducted by Dragovic‐Uzelac et al. [46], it has been established that the presence of phenolic compounds within stone fruits is particularly pronounced during the initial and early phases of their development. However, it is worth noting that the concentration of these compounds tends to decrease as the fruits progress towards maturity. This observation underscores the dynamic nature of phenolic chemical composition within stone fruits as they transition from early growth stages to full ripeness.

### 3.4. Antioxidant Activity

**Figure 3:**
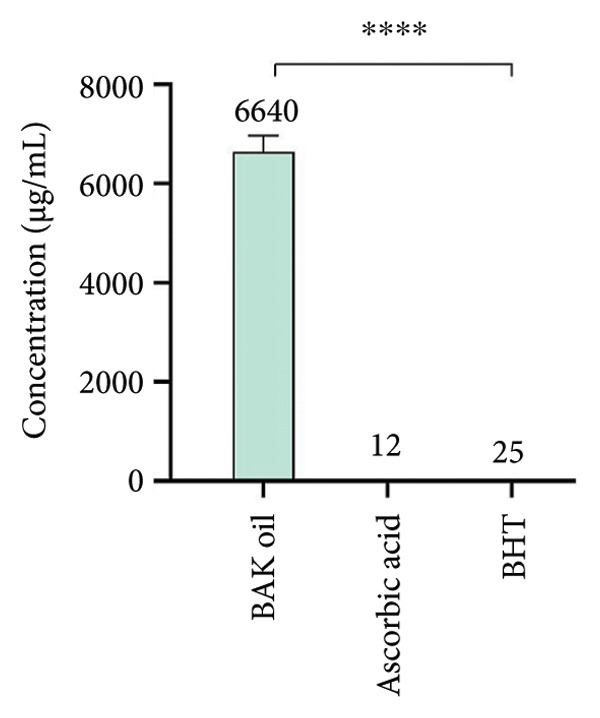
The DPPH scavenging capacity of bitter apricot kernel oil (BAK oil), ascorbic acid, and BHT. Results are significant at ^∗∗∗∗^ *p* < 0.0001.

Several constituents of natural vegetable function as natural antioxidants. In this study, the DPPH scavenging capacity test was performed to investigate the free radical scavenging capacity of our sample that was determined and expressed as IC_50_ values. The results presented in Figure 3 show that the BAK oil exhibited relatively weak DPPH free radical scavenging activity (IC_50_ = 6640 ± 320 μg/mL) compared with the positive controls (*p* < 0.0001), ascorbic acid (IC_50_ = 12 ± 1 μg/mL), and BHT (IC_50_ = 25 ± 1 μg/mL). Although the antioxidant assays confirmed measurable activity of the tested sample, the magnitude of inhibition was considerably lower than that of the standard reference antioxidants. This finding indicates that, while the BAK oil possesses some radical scavenging potential, its effectiveness is limited compared to synthetic or well‐established natural antioxidants. Therefore, its antioxidant capacity should be regarded as moderate to low. This limitation highlights the relevance of considering BAK oil extracts as complementary rather than primary antioxidant agents, and it underscores the need for further investigations to evaluate their potential synergistic effects with other bioactive compounds or their application in contexts where moderate antioxidant activity may still be advantageous. According to Hong‐Lei et al. [47], the Ansu apricot oil exhibited a significant scavenging effect in the DPPH free radicals with an IC_50_ value of 500 μg/mL. Similarly, Tian et al. [48] showed that the oil from white apricot almonds had a strong DPPH scavenging action, with an IC_50_ value of 200 μg/mL. Moreover, Hamid et al. [40] reported that BAK oil exhibited notable antioxidant potential, with an IC_50_ value of 90.44 μg/mL in the DPPH radical scavenging assay. A previous study by Tareen et al. [49] demonstrated that apricot kernels exhibited higher antioxidant activity compared to pomace, with IC_50_ values for DPPH radical scavenging ranging from 24.88 to 98.61 μg/mL in the kernel extracts. Moreover, several studies showed that the apricot kernel extracts have a significant capacity to scavenge free radicals [38, 50].

Regarding the TAC of the studied oil, a considerable value was recorded at 2.21 ± 0.12 mg AAE/g of oil. Our previous reports indicated that BAKs’ aqueous and methanolic extracts exhibited superior TAC values (6.486 ± 0.275 and 5.323 ± 0.826 mg AAE/g, respectively) [43]. In addition, Drogoudi et al. [51] reported that the TAC of 29 different cultivars of apricot and hybrids ranged from 0.026 to 1.858 mg AAE/g. A previous study conducted by Gomaa [52] demonstrated that extracts from sweet apricot kernels exhibited TAC values ranging from 57.78 to 59.53 mg AAE/g. Moreover, Leccese et al.’s research highlighted the dynamic nature of antioxidant capacity in apricots throughout the maturation process. Indeed, a wide variation in TAC values indicated that the antioxidant content changes significantly as the fruit ripens. At the early stage of maturation, the relatively low TAC value (1.14 μmol TE/g FW) may be attributed to the limited exposure to sunlight and the lower photosynthetic activity of the immature fruit. As ripening progresses, TAC values increase markedly, reaching up to 9.93 μmol TE/g FW. This rise reflects the enhanced biosynthesis and accumulation of bioactive compounds particularly polyphenols, flavonoids, and carotenoids which are known to play a central role in antioxidant defense [53].

However, antioxidant activities are not determined solely by the stage of maturity; they are also influenced by factors such as cultivar variation, environmental conditions, cultivation practices, and post‐harvest storage. These parameters can substantially alter the phytochemical profile of apricot kernels and their derived oil, which could explain the variability observed across different studies. Considering these combined effects is essential for accurately evaluating the health‐promoting potential of apricot kernels and their oil, as well as for guiding their optimal use in food, nutraceutical, and cosmetic products. Overall, our findings suggest that BAK oil possesses antioxidant potential, making it a more suitable complementary source rather than a primary one. Therefore, future research should explore strategies to enhance its stability and functionality, such as fortification with natural antioxidants, blending with other oils or investigating synergistic effects with other bioactive compounds to improve its shelf life and broaden its potential applications.

### 3.5. Antimicrobial Activity

BAKs represent a valuable source of bioactive molecules with proven effectiveness against multidrug‐resistant bacterial strains [54]. Its oil has been found to possess antimicrobial activity against various bacterial strains [40]. The findings obtained (Tables 2 and 3) indicated a strong antibacterial activity against Gram‐positive bacteria, with inhibition zones of 10 ± 0.5 and 11.5 ± 0.5 mm for *Staphylococcus aureus* and *Bacillus subtilis*, respectively, and a MIC value of 100 μL/mL for both. In contrast, the Gram‐negative bacteria (*Escherichia coli* and *Pseudomonas aeruginosa*) were found to be more resistant and suffered no effect at the studied concentrations. The obtained results align with those reported by Hamid et al. [40], who found that BAK oil exhibited an antibacterial activity against various bacterial strains, with MIC values ranging from 62.5 to 250 μL/mL. Indeed, the highest antimicrobial activity was observed against *Escherichia coli*, with a growth inhibition zone of 20.3 mm and a MIC value of 62.5 μL/mL. While in a previous study conducted by Singh et al. [35], the highest antimicrobial activity of wild apricot kernel oil was observed against *Salmonella typhimurium*, with a growth inhibition zone of 18 mm and an MIC of 0.150 mg/mL.

**Table 2:** Inhibition zones of bacterial growth by the bitter apricot kernel oil (BAK oil).

Bacterial strains	Inhibition zones (mm)	
	BAK oil	Control
*Escherichia coli*	ND	19.5 ± 0.5
*Pseudomonas aeruginosa*	ND	19 ± 0.3
*Staphylococcus aureus*	10 ± 0.5^∗∗∗∗^	22 ± 0.5
*Bacillus subtilis*	11.5 ± 0.5^∗∗∗∗^	23.5 ± 0.5

*Note:* Results presented as means ± SD. Results are significant at ^∗∗∗∗^ *p* < 0.0001.

Abbreviation: ND, not detected.

**Table 3:** Minimum inhibitory and bactericidal concentrations of bitter apricot kernel oil.

Bacterial strains	BAK oil (μL/mL)		Control (mg/mL)	
	MIC	MBC	MIC	MBC
*Escherichia coli*	ND	ND	0.019	0.038
*Pseudomonas aeruginosa*	ND	ND	0.076	0.076
*Staphylococcus aureus*	100^∗∗∗∗^	ND	0.038	0.038
*Bacillus subtilis*	100^∗∗∗∗^	ND	0.019	0.019

*Note:* Results are significant at ^∗∗∗∗^ *p* < 0.0001.

Abbreviations: BAKs, bitter apricot kernels; MBC, minimum bactericidal concentration; MIC, minimum inhibitory concentration; ND, not detected.

The study carried out by Tia et al. [48], concerning the antimicrobial activity of white almond oil against 10 microbial strains showed that the largest inhibition zone was observed against *B. subtilis* (35.04 ± 0.32 mm), while the smaller inhibition zone was observed against *P. citrinum* (15.05 ± 0.11 mm), and the most significant MIC value was recorded against *E. coli* (0.2 mg/mL). Moreover, several studies conducted previously highlighted the efficacy of the extracts obtained from BAKs as an antibacterial agent [52, 55, 56].

The observed antibacterial efficacy of our sample can be attributed to the presence of lipids, particularly free fatty acids, known for their potent antimicrobial properties. These compounds exhibit the capability to effectively eliminate a wide range of microorganisms, including Gram‐negative and Gram‐positive bacteria, fungi, and enveloped viruses upon contact [57]. The antimicrobial efficacy of fatty acids is due to their detergent‐like properties, which interact with bacterial cell membranes. These properties solubilize membrane lipids and proteins, causing structural disruptions that impair essential metabolic processes like the electron transport chain and oxidative phosphorylation. These membrane alterations may additionally impede nutrient absorption, hinder enzymatic function, and induce the formation of peroxidation byproducts with potential toxic effects [58].

Although Gram‐positive bacteria possess a thicker peptidoglycan layer, they lack an outer membrane [59]. As a result, their cytoplasmic membrane is more directly exposed and accessible to lipophilic compounds such as fatty acids and essential oils, which can insert into and disrupt the phospholipid bilayer, leading to measurable inhibition zones (10–11.5 mm) and MIC values (100 μL/mL). In contrast, Gram‐negative bacteria are protected by an additional outer membrane that serves as a selective permeability barrier, limiting the penetration of hydrophobic molecules [60] and explaining the absence of activity at the tested concentrations. This structural distinction is widely recognized as the main reason why Gram‐positive bacteria are generally more susceptible. Additional mechanisms, such as efflux pumps and differences in membrane lipid composition, may also contribute to the comparatively higher resistance of Gram‐negative species [61–63].

Although the present study highlights the nutritional value and biofunctional activities of Moroccan BAKs and their oil, it is essential to recognize the potential safety risks associated with their consumption. These kernels are known to contain amygdalin, a cyanogenic glycoside that can release hydrogen cyanide (HCN) upon hydrolysis [64]. Excessive intake of amygdalin‐rich kernels has been associated with acute cyanide poisoning, and regulatory agencies in several countries have issued strict guidelines or restrictions on their dietary use. It is noteworthy, however, that the oil extracted from BAKs generally contains negligible amounts of amygdalin, as this compound is primarily water‐soluble and concentrated in the kernel cake rather than the lipid fraction [65–67].

A previous study conducted by Viorica‐Mirela et al. [68] reported amygdalin concentrations of ∼7–24 mg/kg in kernels, whereas it was not detected in the oils. In addition, Jin et al. [69], reported that the amygdalin content in bitter apricot oil was detected at levels up to 5 mg/kg, which is considered safe for consumption.

Similarly, the study by Pavlović et al. [70] reported that cold‐pressed oil extracted from apricot kernels contained small amounts of amygdalin (approximately 0.40 mg/g). This suggests that limited quantities of amygdalin may migrate into the oil during extraction, although the concentrations remain substantially lower than those found in the kernels themselves.

Furthermore, El Hajjaji et al. [71] reported that the aqueous extract of Moroccan BAKs was nontoxic in mice, with a median lethal dose (LD_50_) exceeding 6 g/kg and only minor hematological and biochemical alterations observed at the highest subacute dose (1 g/kg bw), thus providing valuable insights into its overall toxicity profile. Similarly, another study demonstrated that oral administration of the aqueous extract of apricot seeds at doses of 1–2 g/kg body weight per day for 48 days did not result in any significant renal or hepatic toxicity [72]. Nevertheless, further investigation into the residual amygdalin content of BAK oil is required to confirm its safety for food and pharmaceutical applications.

## 4. Conclusion

This research reinforces the growing body of evidence highlighting the diverse biological potentials of BAK oil, which hold significant promise for enhancing human health. The detailed analysis revealed the presence of significant concentrations of essential minerals in the kernels. The investigated oil has a remarkable concentration of phenolic compounds, including polyphenols and flavonoids. Furthermore, it demonstrated notable capabilities in scavenging free radicals and showcased significant antibacterial properties. Considering these compelling findings and extensive observations, BAK oil emerges as a highly beneficial asset with potential applications across the medical, cosmetic, and health supplement domains. Its multifaceted properties position it as a valuable resource for various health‐enhancing purposes and product formulations.

## Disclosure

All authors read and approved the final version of this manuscript.

## Conflicts of Interest

The authors declare no conflicts of interest.

## Author Contributions

Mohamed Amine El Hajjaji: investigation, methodology, formal analysis, data curation, and writing–original draft; Kawtar Fikri‐Benbrahim: conceptualization, supervision, validation, and writing–review and editing; Najoua Soulo: methodology, formal analysis, and data curation; Younesse El‐Byari: investigation, methodology, formal analysis, and data curation; Zineb Benziane Ouaritini: conceptualization, supervision, and validation.

## Funding

No external funding was received for this research.

## Data Availability

Data are available on request from the authors.
